# Critical Success Factors of Safety Program Implementation in Construction Projects in Iraq

**DOI:** 10.3390/ijerph18168469

**Published:** 2021-08-11

**Authors:** Mohanad Kamil Buniya, Idris Othman, Riza Yosia Sunindijo, Ghanim Kashwani, Serdar Durdyev, Syuhaida Ismail, Maxwell Fordjour Antwi-Afari, Heng Li

**Affiliations:** 1 Department of Civil & Environmental Engineering, University Technology PETRONAS, Seri Iskandar 32610, Malaysia; idris_othman@utp.edu.my; 2 School of Built Environment, The University of New South Wales, Sydney, NSW 2052, Australia; r.sunindijo@unsw.edu.au; 3 Division of Engineering, New York University Abu Dhabi, P.O. Box 129188, Abu Dhabi 51133, United Arab Emirates; gak289@nyu.edu; 4 Department of Engineering and Architectural Studies, Ara Institute of Canterbury, Christchurch 8011, New Zealand; durdyevs@ara.ac.nz; 5 Razak Faculty of Technology and Informatics, Universiti Teknologi Malaysia, Kuala Lumpur 541000, Malaysia; syuhaida.kl@utm.my; 6 Department of Civil Engineering, College of Engineering and Physical Sciences, Aston University, Birmingham B4 7ET, UK; m.antwiafari@aston.ac.uk; 7 Department of Building and Real Estate, The Hong Kong Polytechnic University, Room No. ZS734, Hung Hom, Kowloon, Hong Kong; heng.li@polyu.edu.hk

**Keywords:** safety program, construction, critical success factors, PLS-SEM, Iraq

## Abstract

The construction sector is recognized as one of the most dangerous industries in the world. The situation is worsening in Iraq, as a result of a lack of attention to safety in the building industry and the poor implementation of safety programs. This research aims to identify the critical safety factors (CSFs) of safety program implementation in the Iraqi construction industry. The CSFs were first identified from a review of literature before being verified by construction practitioners, using semi-structured interviews. A questionnaire, based on the verified CSFs, was distributed to construction practitioners in Iraq. Exploratory factor analysis (EFA) was used to analyze the quantitative data, and the results show that the CSFs can be categorized into four constructs: worker involvement, safety prevention and control system, safety arrangement, and management commitment. Following that, partial least square structural equation modelling (PLS-SEM) was executed to establish the connection between safety program implementation and overall project success. The result confirms that safety program implementation has a significant, positive impact on project success. This article contributes to knowledge and practice by identifying the CSFs for implementing safety programs in the Iraqi construction industry. The successful implementation of a safety program not only improves safety performance, but also helps to meet other project goals.

## 1. Introduction

Construction is an important sector that provides necessary infrastructure and facilities, which contribute to the wellbeing of society [1]. Strong linkages exist between the construction sector and other sectors, which, by and large, have proven to be the impetus for the growing influence of the construction sector in the economic development around the world [2]. The importance of the sector is proven by its rapid growth in recent years, a growth which experts expect to continue, despite the impact of the COVID-19 pandemic [3]. The construction industry is also a large employer of labor, thereby promoting economic and social development.

Unfortunately, despite its significance, the industry has poor safety performance [4]. Early statistics revealed that about 7% of workers worldwide were engaged in the construction industry, but they represent 30–40% of fatalities across industrial sectors [5,6]. In South Korea, construction recorded the highest proportion (25.3%) of deaths in the workplace [7,8]. It is the third-highest in the US and the fourth-highest in Australia [9]. Researchers, in various studies, have shown that the fatality rate in the construction industry is high [10]. The negative impact of poor safety performance is represented by the affects on time, cost, and productivity [11]. Therefore, there is a need for the implementation of an effective safety program to improve safety performance.

The Iraqi construction industry also faces the same challenge. The rapid growth of the industry is plagued by a lack of effort towards safety, resulting in poor accident prevention [12,13]. The Iraqi construction industry needs to implement an effective safety program, to develop a safety culture and improve its safety performance [14]. The effective implementation of a safety program, however, requires an improved understanding of the elements and success factors of the safety program and their impacts on project performance. Furthermore, management commitment and the involvement of key stakeholders are critical for providing adequate resources to facilitate the immediate success of the program [15,16,17].

It follows, based on the foregoing, that decisively resolving the safety problem from the root by implementing a proactive safety program in the Iraqi construction industry is a matter of urgency. There have been no studies that focus on identifying the critical success factors (CSFs) that affect safety program implementation in building projects in Iraq. The use of the partial least square structural equation modeling (PLS-SEM) to develop a mathematical model, a technique that has never been explored (in relation to safety implementation), helps establish the relationships between the CSFs and project performance. To fill these gaps, this study aims to examine the effect of CSFs on safety program implementation, by using PLS-SEM in building projects in Iraq.

## 2. Success Factors of Safety Program Implementation

Safety programs can be defined as the actions of people to avoid illnesses and injuries in the workplace [18]. Anton [19] stated that a safety program should involve both the monitoring and control of the environment, workplace, facilities, practices, and employees, in order to minimize accidents, injuries, and losses at work. As explained by Rowlinson [16], a safety program is a means of reducing potentially dangerous behavior that can lead to an accident, as well as recognizing and reporting safety issues and injuries.

CSFs are outcome-based elements that can ensure success within the organization [20]. In the safety context, CSFs involve activities, resources, and behavior needed to successfully implement a safety program [21]. The following 22 CSFs have been taken into account, based on the results of previous studies concerning safety program implementation, especially in developing countries (Table 1).

There are several advantages associated with the effective implementation of safety programs [60,61], such as decreasing accident-related costs, reducing absenteeism and turnover, increasing productivity, and generating better worker morale [62,63]. Oliveira et al. [64] added that organizations that implement safety programs enhance work quality, build reputations, improve employee collaboration, and increase profits. Rowlinson [65] explained that the objectives of the implementation of safety programs should be the prevention of improper and insecure conduct, the reporting of safety risks and hazards, and the documentation and management of incidents. Therefore, this research hypothesizes that CSFs have a significant effect on project success in the Iraqi construction sector for safety program implementation.

## 3. Research Method

**Figure 1:**
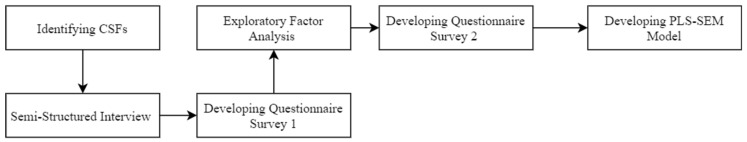
Research process.

A mixed method approach was used in this study, as illustrated in the research process (Figure 1). A set of CSFs was identified from the literature, as discussed earlier (Table 1). Sixteen semi-structured interviews were conducted to review and adjusted the variables for developing a questionnaire [66]. Initially, 21 CSFs were drawn from the literature. Technology was added, based on the input from the interview participants, bringing the total number of CSFs to 22.

A pilot study (Questionnaire 1) was then conducted using a list of safety program CSFs among building professionals who have appropriate industry experiences [67]. Data collected from the pilot study were analyzed using Exploratory Factor Analysis (EFA), performed to check for the extensiveness and clarity of the CSFs of the safety program. The outcomes of the EFA confirm the relevance of the 22 CSFs and they were used in the primary survey (Questionnaire 2).

### 3.1. Model Development

The use of “Partial Least Square Structural Equation Modeling (PLS-SEM)” has received considerable interest from various fields of study, especially in business and social sciences [68,69]. To model the safety program CSFs, Partial Least Square was used to analyze the data for its excellent predicting purposes [70]. PLS-SEM is primarily a causal modelling approach, designed to maximize the explained variance of dependent latent constructs with normal, smaller sample sets [71].

#### 3.1.1. Measurement Model

This section demonstrates the relationship between the items and their original latent structure [72]. The following sections discussed the measurement model’s convergent and discriminant validity.

##### Convergent Validity

Convergent validity refers to the degree of agreement between two or more measurements (CSFs) of the same construct (group). [73]. It is regarded as a sub-set of the construct’s validity. When using the PLS model, three tests may be used to assess the convergent validity of the measured constructs [74]: “Cronbach’s alpha” (α) “composite reliability” ($\rho_{c}$), and “average variance extracted” (AVE). A Cronbach’s alpha and $\rho_{c}$ value of 0.7 was proposed by Nunnally and Bernstein as the threshold for ‘modest’ composite reliability [74], while values above 0.60 for exploratory studies were appropriate [75]. Finally, AVE was carried out as the last test. It is a normative measure for evaluating the convergent validity of constructs in a model, with values greater than 0.50 suggesting a reasonable convergent validity [75].

##### Discriminant Validity

“Discriminant validity” signifies the tested phenomenon as empirically distinct and indicates that any measurements that are not detected in the phenomenon are being tested in the SEM [76]. Campbell and Fiske [77] argued that similarities between different measures should not be too high for discrimination to be established.

##### Structural Model Analysis

This analysis produces a priority model using SEM for the CSFs of VM. Amaratunga et al. [78] stated that SEM is a useful method for dealing with errors in variables. This technique can be applied to oversimplify a complex decision-making process [79]. The path coefficients between the observed coefficients should be specified to complete the analysis for this study. The hybothesis in this study shows the causal relationship (path relationship) between “α” (CSFs of safety program constructs) and “μ” (CSFs of safety program). The structural relationship between the α, μ, and €1 formula shows the inner relationship that exists in the linear equation, as shown below [80,81]:

µ = βα + ᾱ1(1)

where (β) is the path coefficient connecting the CSFs of safety program constructs and (ᾱ1) is the expected residual variance at the structural level. β is the standardized regression’s uniform weight, equivalent to a multiple regression model’s β weight. It is a sign that there is consistency in the model forecasts and is statistically relevant.

The next step is calculating the significance of the path coefficient. Confirmatory factor analysis (CFA) was used to calculate the coefficients’ standard errors using the bootstrapping technique with Partial Least Square. Henseler et al. [68] suggested 5000 subsamples, grounded for proposition testing of the t-statistics. For the PLS Model, four structural equations were generated for safety program CSFs constructs, describing the constructs’ inner relations and the Equation (1).

## 4. Data Collection and Case Study

### 4.1. Interviews

Based on the method proposed by Sanders [82] and Hesse-Biber [83], it was considered that ten interviews were sufficient for this type of research. Therefore, fifteen experts were chosen, based on their experience, education, and location using a “purposeful sampling” approach. This method helps achieve the research aims by controlling the degree of difference between interviewees [84].

Given the different roles of building specialists in construction projects, the interviewees had a wide range of backgrounds. It should be noted that this study employed a technique identified as the “abductive approach” [85]. This method has been used to establish a hypothetical framework and analysis, based on the purpose and objectives of the study, by using literature. Previous studies were used to establish a theoretical foundation for creating the inquiries and analytic methodologies in this method. [86]. Previous studies were used to produce the theoretical structures (safety programs CSFs) used in the current study. This method used the structural standards needed to evaluate the current hypotheses and generate new theories. Subsequent interviews enhanced the method and expanded it. As a result, the abduction approach was employed in this study to re-examine and assess the reality and contemporary frameworks of the CSFs build, in a particular context.

Subsequently, the interviewed experts consented to a more systematic safety program adoption strategy that should guide the direct adoption of safety programs in construction projects. Table 1, presented earlier, shows the classification of the safety program CSFs into four categories. Several other CSFs were also changed, and one variable (technology) was added to the list. The updated CSFs were used to create the EFA study questionnaire.

### 4.2. Pilot Study (Questionnaire 1)

To collect quantitative data for the EFA, pilot research was conducted by sending 200 questionnaires to Iraqi building specialists. The EFA results approved the category and all the CSFs in Table 1.

### 4.3. Main Survey (Questionnaire 2)

The EFA results were used to produce the primary survey (Questionnaire 2) to analyze safety program CSFs in Baghdad, Anbar, Basra, and Erbil. This survey consists of three key sections: the respondent’s demographic profile, the safety program CSFs (Table 1), and the questionnaire. Using a 5-point Likert scale, ranging from 1 (very small) to 5 (very high), respondents were asked to measure the levels of implementation and effectiveness of the CSFs.

Kline [87] found that sample sizes of 200 or larger were needed for a complex path model, while Yin [88] found that the sample size of the SEM should be higher than 100. A total of 223 respondents, out of 300 respondents who were personally approached (self-administered), participated in the survey, translating into a response rate of about 67.5%. Twenty-seven responses were incomplete and discarded, bringing the total valid responses to 196. For this analysis, the response rate was considered acceptable [89,90].

### 4.4. Demographic Profiles

**Figure 2:**
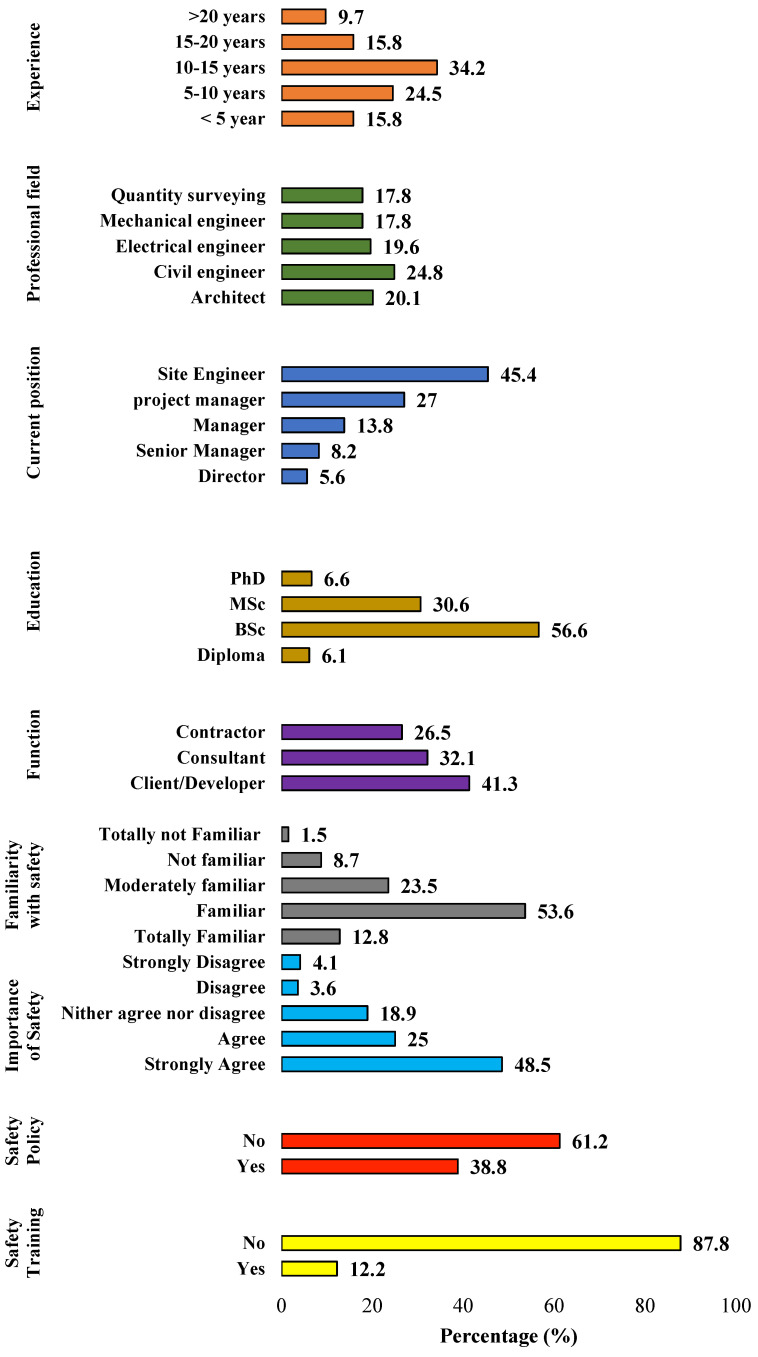
Demographic Profile.

Figure 2 presents the respondents’ demographic profile, including experience, position, level of education, organization type and function, familiarity with safety, and safety training participation.

The effective implementation of safety programs is affected by the level of awareness and knowledge of the project team [21]. Figure 2 shows that the majority of the respondents (87.8%) did not have safety training, indicating that the levels of safety knowledge among the respondents might be below expectations. The non-existence of a safety policy is concerning because it demonstrates an apparent lack of safety commitment in the Iraqi construction industry. Furthermore, the results show that only about 65% of the respondents were familiar with safety. Considering that more than 60% of the respondents work for public organizations, it can, therefore, be concluded that government efforts and regulations to improve safety in the industry are inadequate.

## 5. Results

### 5.1. Factor Analysis

The factor structure of the 22 CSF items of a safety program was determined by the EFA approach. The “Kaiser-Meyer-Olkin”, which measures the sampling adequacy, was 0.771 above the suggested value of 0.6, while the Bartlett’s test of sphericity was significant (χ2 (231) = 2022.72, *p* < 0.05). Both indicators show that factor analysis is appropriate for analyzing the data. To allow the enclosure of the elements in the factor analysis, we ensured that each diagonal of the anti-image correlation matrix was greater than 0.5. Estimates of the variance, or rather initial commonalities, in each variable were accounted for by all components, and the small value “<0.3” indicates variables do not fit with the factor. The loading factors were all above 0.5, and all initial communalities were above the threshold (Table 2).

Five factors have been extracted from the 22 items, following the analytical executions with eigenvalues greater than 1. The eigenvalues and total variance are well-explained by the five factors (68.83%), as presented in Table 3. On running Varimax rotation, the first factor linked to “safety prevention and control system” explained 20.801% of the variance, whereas the second factor, “worker involvement”, had 14.925% of the variance. The third factor, “safety arrangement” can be explained by 13.788% of the variance, and the fourth component, “safety commitment”, explained 12.806% of the total variance. The last component only had a single item (SPCS7), which originally belonged to other components; therefore, this item was excluded.

Researchers tested the reliability of the questionnaire after EFA by using the alpha of Cronbach. [76]. Table 4 shows that the Cronbach’s alpha values range from 0.843 and 0.870. All values fall well above the minimum threshold (0.7), indicating that the questionnaire and the components are reliable.

### 5.2. CSFs of Safety Program Implementation—Structural Equation Model

#### 5.2.1. Measurement Model

The PLS algorithm analysis approach utilizes an active model to establish the relationship existing between the exogenous variable and its corresponding latent variable [91]. In this stage, the assessment includes convergent validity, discriminatory validity, and the internal reliability of the model. The convergent validity gives the degree to which correlation between two or more variables of the same group stands [73] and it contains three tests, namely average variance extracted (AVE), composite reliability (CR), and Cronbach’s alpha [74]. The acceptable level of reliability is 0.7 [71,92], the minimum value of the AVE is 0.5 [70,74,93], and the minimum CR value is 0.7 [75]. Table 5 shows that all the values meet the minimum requirements.

The discriminant validity is the second test in the measurement model, which was conducted using the PLS algorithm. The root square of the AVE for each construct can be applied to the correlations of a construct, along with all other constructs, to determine the discriminant validity. Discriminant validity would suggest that a construct is special and fully capable of expressing any phenomena which are not represented in the model by other constructs. This study used the cross-loading criterion to estimate the discriminant validity. The outward loading on the linked constructs should exceed all its loading on other constructs. The cross-loading results of the constructs demonstrate a high degree of unidimensionality (Table 6).

#### 5.2.2. Second-Order Test/Path Analysis

The second order for CSFs was formative latent variables. The significance of the path coefficients was evaluated with the bootstrap tool. It is also necessary to establish the collinearity of the formative items, which prompt the evaluation of “variance inflation factor” (VIF) value. Accordingly, all the VIF were less than 3.5, indicating that the components are self-sufficiently contributed to the higher-order construct.

Table 7 shows the resulting CSFs with four subscales, namely, safety prevention and control (β = 0.441, *p*-value < 0.001), worker involvement (β = 0.252, *p*-value < 0.001), safety arrangement (β = 0.236, *p*-value < 0.001), and safety commitment (β = 0.215, *p*-value < 0.001). The second order results approved the four subscales have a significant effect on safety program implementation.

Path analysis is a technique used to assess the relationship between the constructs and to examine the research hypotheses. Table 8 shows that the CSFs have a positive and significant influence (β = 0.546, *p* < 0.001) on project success.

After reliability and validity were established, the evaluation criteria for PLS-SEM results were the coefficients of determination (R^2^ values). The coefficient of determination is a measurement of how much of the variance in an endogenous construct is explained by its predictor constructs. The R^2^ line refers to how much of the variance independent variables is explained by independent variables. Thus, a larger R^2^ value rises the predictive capability of the structural model.

**Figure 3:**
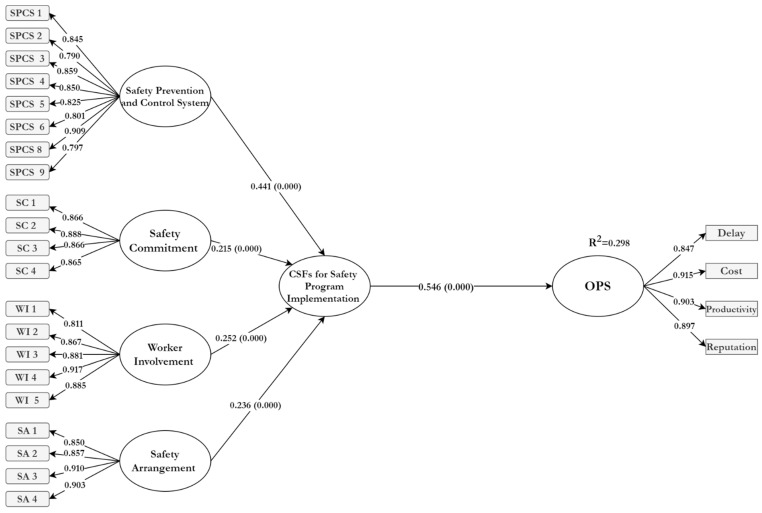
Structural Model.

Figure 3 shows the results of $R^{2}$, for overall project success (OPS) in this model, was 0.298. This means that the CSFs can contribute 29.8% to the OPS.

## 6. Discussion

The Iraqi construction industry has poor safety performance and implementing a safety program is a way of address this challenge, so that safety can be considered as an integral aspect in construction projects. Understanding the CSFs of safety program implementation is crucial to ensure the success of the program and, eventually, the overall success of the project.

The successful implementation of a safety program frequently requires a wide range of knowledge (e.g., CSFs affecting safety programs), as well as an acceptable level of safety program comprehension from various stakeholders. We infer that Iraqi building experts are more aware familiar with the advantages of safety with more than 53%. This implies that safety awareness is relatively commensurate with that of other emerging countries [11].

Therefore, this study examines the impact of the CSFs of safety program implementation on project success. The finding of this research confirms that the CSFs have a significant relationship with OPS [path coeffect (β) = 0.546] and they can contribute up to 29.8% to OPS. This study also confirms various CSFs to achieve success in safety program implementation. The finding of this study is in line with previous studies, by approving the effect of CSFs on OPS mathematically [15,21].

The following subsection discusses the CSFs clusters, after completing EFA analysis by using SEM-PLS.

### 6.1. Worker Involvement

Worker involvement is an essential prerequisite for the implementation of a safety program. The path coefficient above (0.215) confirms this factor significantly influences safety program implementation. The items that represent this factor include personal attitude, motivation, safety meetings, safety committee, and continuing participation. The attitudes of workers (including project personnel) toward safety is influenced by their motivation. In this case, one of the ways to strengthen their motivation is by continuously engaging them to fully participate in safety activities, such as regular safety meetings and inspections.

### 6.2. Safety Prevention and Control System

The safety prevention and control dimensions include several items, such as enforcement scheme, dedicated health and safety personnel, training, equipment and maintenance, personal competency, program evaluation, pre-task planning for safety, inspection, and technology. This factor strongly affects safety program implementation, as indicated by the path coefficient (0.454). An effective enforcement scheme is essential to ensure workers and staff strictly follow safety regulations and rules [56]. A study found that an effective enforcement mechanism is effective in reducing safety violations [37]. While some activities on construction sites are deemed to be complex and hazardous, an effective control system can reduce the level of risks and prevent accidents, particularly in developing countries where safety risk management and enforcement are lacking [21]. Aksorn and Hadikusumo [21] and Al Haadir and Panuwatwanich [15] argued that this control system should be supported by safety training to improve safety awareness and equip workers with necessary safety knowledge and skills. According to Saurin et al. [94], pre-task planning can improve the overall implementation of the safety management system by making workers much aware of daily risks when performing particular construction activities. Finally, technology also has a positive impact on the safety program. Initially, this factor was not included in previous studies and was added by Iranian construction experts during the interview process. Technology can facilitate effective communication and be used to monitor construction activities remotely, which has the potential to improve safety performance.

### 6.3. Safety Arrangement

This factor includes communication, allocation of authority and responsibility, appropriate resource allocation, and hazard identification. The path coefficient (0.215) shows that this factor significantly influences the implementation of a safety program. Safety arrangement is irreplaceable in safety program implementation because it facilitates safety communication, thus helping everyone to follow safety procedures and be aware of their safety responsibilities [46]. Furthermore, the successful implementation of a safety program is likewise subject to the availability of resources, including human resources, money, adequate duration, communication channels, and equipment [52].

### 6.4. Management Commitment

This factor affects safety program implementation significantly, as demonstrated by a path coefficient of 0.210. Among the elements that contribute to this factor are management support, clear and realistic goals, and safety policies. Management commitment is a foundational factor that ensures the success of safety program implementation [95]. The top management should demonstrate their commitment by consistently communicating to all that safety is the priority in the workplace. They should also manifest their commitment by participating in safety activities and providing adequate resources. This study again confirmed that management commitment is essential to controling and implementing safety programs successfully, which is in line with Huang and Hinze [96] and Hassan et al. [97].

## 7. Conclusions

Safety programs have been introduced in many countries to reduce injuries and fatalities in the workplace, minimize accident-related costs, and improve reputation in the construction industry. Safety performance remains poor in Iraq, despite the importance of the construction industry to the country’s economic growth. The implementation of a safety program is seen as a key initial step to address this challenge. This study has identified the CSFs that affect safety program implementation in construction projects in Iraq. The effects of these CSFs on safety program implementation have been established using data collected from 196 industry practitioners in Iraq. A model was developed and validated empirically by using PLS-SEM after the CSFs were grouped into factors using EFA. The final CSFs (21 in total) are categorized into four dimensions, namely worker involvement, safety prevention and control system, safety arrangement, and management commitment. A PLS-SEM was then used to validate the EFA result. Analysis of the data shows a significant influence of CSFs on the safety program implementation. Indeed, this empirical evidence of the relationship existing between CSFs and safety program implementation contributes to the body of knowledge in the Iraqi construction industry and, to a broader extent, the safety in construction projects. This study has also confirmed that a safety program implementation, facilitated by the CSFs, can contribute 28.9% to OPS. This finding can help decision-makers and stakeholders in their policy planning, development, and implementation of safety programs for improving the construction industry’s safety performance. Furthermore, the outcome of the study laid down a foundation for new research by confirming the positive influence of CSFs on safety management. This study, for the first time focusing on Iraq, provides a foundation for future studies about the impacts of CSFs on safety program implementation in developing countries.

There are some study limitations worth discussing. First, the present research focused on Iraq, as a case study, and collected data from four cities (Bagdad, Anbar, Basra, and Erbil). Future studies can use another case study context to confirm the research findings. Second, 16 interviews were conducted to identify the CSFs. Although data maturation seemed to be achieved during the analysis, a larger sample size may reveal new factors. Finally, future research can consider the impacts of demographic profiles, such as locations, company size, and positions on the successful implementation of a safety program. Different approaches may be tailored, based on the demographic factors investigated.

## Figures and Tables

**Table 1:** Critical success factors.

Groups/Items		Code	Description	References
**“Worker Involvement”**				
1	Personal attitude	WI1	Attitude is the ability to positively or negatively react to certain person, objects, or conditions and is typically established through experience. If the positive attitudes of employees (toward safety) are reinforced, a successful safety program can be achieved.	[21,22]
2	Motivation	WI2	To promote safety, all personnel should be motivated to perform their responsibilities safely, via the possibility of achievements and recognitions for additional responsibilities, rewards, and personal growth.	[23,24,25]
3	Safety meeting	WI3	Regular safety meetings must be organized to review safety performance.	[26]
4	Safety committee	WI4	A committee (which consists of supervisors, managers, and workers) to manage safety activities, such as meetings and inspections.	[20,24,27,28,29]
5	Continuing participation	WI5	Employee involvement is very important for a successful safety program. The worker should have opportunities to participate in implementing the safety program.	[24,30,31,32,33]
**“Safety prevention and Control system”**				
1	Enforcement scheme	SPCS1	To ensure the safety rules and regulations are followed, there must be an effective enforcement system in place.	[34,35,36]
2	Appropriate supervision	SPCS2	There should be appropriate supervision to protect workers from workplace hazards. Successful supervision can make the workplace safe by collaborating with workers and listening to them. This encourages workers to follow rules and regulations and promotes collaboration in addressing safety problems.	[37]
3	Training	SPCS3	Employees should receive adequate safety training to improve their safety knowledge, skills, attitudes, and behaviors.	[22,38,39]
4	Equipment and maintenance	SPCS4	Appropriate equipment and regular maintenance are required to create a safe working environment.	[38,39]
5	Personal competency	SPCS5	Having the right person on the right job is crucial to have a successful safety program implementation. This encourages people to do the right thing, at the right time, by using experience and skills to identify hazards and make the right decisions to reduce risks.	[22,38,40]
6	Program evaluation	SPCS6	Safety program implementation should be evaluated periodically to determine its success in meeting the objective and goals. If the safety program does not meet the goals, the evaluation can identify areas for improvement.	[24,41]
7	Pre task planning for safety	SPCS7	Safety meetings will be held shortly before work begins to ensure that everyone is aware of the hazards and risks involved.	[14,42,43]
8	Site system inspection	SPCS8	Safety inspection of site to identify hazards and violations of safety regulations and policies.	[17,20,29,44,45]
9	Technology	SPCS9	Adopting technologies to enhance safety.	[13]
**“Safety Management”**				
1	Communi-cation	SM1	Effective safety communication between all levels in the project and organization. Workers can report unsafe conditions and acts.	[24,46]
2	Allocation of authority and responsibility	SM2	Safety authorities and responsibilities are delegated appropriately. Everyone is also accountable for safety.	[19,24,46]
3	Appropriate resource allocation	SM3	Adequate resources, including time, money, staff, information, and methods are provided.	[24,41,47,48]
4	Hazard identification	SM4	Reviewing construction plan, methods, materials, and equipment to identify hazards.	[20,28,49,50]
**“Safety Commitment”**				
1	Management support	SC1	Management should demonstrate safety commitment by allocating resources, having a safety policy, participating in regular safety meetings, and visiting work sites.	[24,46,48,51,52]
2	Teamwork	SC2	The safety program succeeds when all levels of staff are engaged and realize that safety is everyone’s responsibility. The goals of safety program can be achieved when all employees work collaboratively in implementing the program.	[53,54,55]
3	Clear and realistic goals	SC3	Safety goals should provide a clear direction for all staff to reach desired results. By completing the goals, safety performance can be measured.	[24,56,57]
4	Safety policies	SC4	Having a safety policy and safety regulations to guide practices and develop safety culture.	[15,17,27,45,58,59]

**Table 2:** Communalities of 22 CSF items.

Item	Commonalities	Item	Commonalities
**WI1**	0.610	**SPCS7**	0.488
**WI4**	0.729	**SPCS8**	0.768
**WI3**	0.612	**SM3**	0.835
**SM1**	0.639	**SC2**	0.818
**SM2**	0.779	**SPCS5**	0.534
**SPCS1**	0.586	**SPCS9**	0.677
**SPCS2**	0.646	**SPCS3**	0.548
**SM4**	0.813	**SPCS6**	0.775
**SPCS4**	0.712	**WI2**	0.679
**SC4**	0.764	**SC3**	0.757
**SC1**	0.711	**WI5**	0.665

**Table 3:** Factor loadings for 22 CSF items (N = 150).

	Component				
	1	2	3	4	5
**SPCS6**	0.820				
**SPCS4**	0.769				
**SPCS9**	0.767				
**SPCS8**	0.767				
**SPCS2**	0.739				
**SPCS3**	0.711				
**SPCS1**	0.687				
**SPCS5**	0.546				
**WI4**		0.789			
**WI2**		0.782			
**WI1**		0.762			
**WI3**		0.746			
**WI5**		0.702			
**SM3**			0.896		
**SM4**			0.870		
**SM1**			0.777		
**SM2**			0.618		
**SC2**				0.883	
**SC4**				0.808	
**SC3**				0.738	
**SC1**				0.691	
**SPCS7**					0.592 *
**Eigenvalues**	4.576	3.283	3.033	2.817	1.434
**% of Variance**	20.801	14.925	13.788	12.806	6.518

* These items were excluded due to cross-loading.

**Table 4:** Reliability Analysis.

Factor Name	Reliability
Safety Arrangement	0.866
Management commitment	0.847
Safety prevention and control system	0.870
Worker Involvement	0.834

**Table 5:** Measurement model results.

Construct	Item	Outer Loadings		Cronbach’s Alpha	CR	AVE
		Initial	Modified			
**Safety Commitment**	SC1	0.866	0.866	0.894	0.926	0.759
	SC2	0.888	0.888			
	SC3	0.866	0.866			
	SC4	0.865	0.865			
**Safety Management**	SM1	0.850	0.85	0.903	0.932	0.775
	SM2	0.857	0.857			
	SM3	0.910	0.91			
	SM4	0.903	0.903			
**Safety prevention and Control system**	SPCS1	0.845	0.845	0.938	0.949	0.698
	SPCS2	0.790	0.79			
	SPCS3	0.859	0.859			
	SPCS4	0.850	0.850			
	SPCS5	0.825	0.825			
	SPCS6	0.801	0.801			
	SPCS8	0.909	0.909			
	SPCS9	0.797	0.797			
**Worker Involvement**	WI1	0.811	0.811	0.921	0.941	0.762
	WI2	0.867	0.867			
	WI3	0.882	0.882			
	WI4	0.917	0.917			
	WI5	0.885	0.885			

**Table 6:** Measurement Model Cross-Loading.

Items	Safety Management	Safety Prevention and Control System	Safety Commitment	Worker Involvement
**SM1**	**0.85**	0.58	0.51	0.53
**SM2**	**0.86**	0.65	0.63	0.56
**SM3**	**0.91**	0.59	0.57	0.50
**SM4**	**0.90**	0.67	0.61	0.59
**SPCS1**	0.67	**0.85**	0.55	0.60
**SPCS2**	0.60	**0.79**	0.58	0.56
**SPCS3**	0.59	**0.86**	0.62	0.58
**SPCS4**	0.59	**0.85**	0.60	0.60
**SPCS5**	0.57	**0.83**	0.55	0.52
**SPCS6**	0.62	**0.80**	0.55	0.62
**SPCS8**	0.63	**0.91**	0.62	0.60
**SPCS9**	0.47	**0.80**	0.55	0.53
**SC1**	0.61	0.66	**0.87**	0.59
**SC2**	0.64	0.61	**0.89**	0.52
**SC3**	0.50	0.55	**0.87**	0.55
**SC4**	0.54	0.58	**0.87**	0.57
**WI1**	0.55	0.62	0.53	**0.81**
**WI2**	0.54	0.62	0.63	**0.87**
**WI3**	0.48	0.54	0.51	**0.88**
**WI4**	0.54	0.58	0.56	**0.92**
**WI5**	0.59	0.64	0.55	**0.89**

Bold indicates the largest value in column.

**Table 7:** Test of second-order formative models using bootstrapping.

Construct	β	SE	T Statistic	*p*-Value	VIF
**CSF -> Safety Commitment**	0.215	0.010	22.136	<0.001	2.289
**CSF -> Safety Management**	0.236	0.009	25.261	<0.001	2.335
**CSF -> Safety prevention and Control**	0.441	0.013	33.819	<0.001	2.862
**CSF -> Worker Involvement**	0.252	0.011	22.895	<0.001	2.164

**Table 8:** Hypotheses and relative paths for the model.

Path	β	SE	T Value	*p* Values
**CSF -> Project Success**	0.546	0.059	2.286	0.001

## Data Availability

The data presented in this study are available on request from the corresponding author. The data are not publicly available, due to privacy and confidentiality issues.
